# Dme-Hsa Disease Database (DHDD): Conserved Human Disease-Related miRNA and Their Targeting Genes in *Drosophila melanogaster*

**DOI:** 10.3390/ijms19092642

**Published:** 2018-09-06

**Authors:** Guanyun Wei, Lianjie Sun, Shijie Qin, Ruimin Li, Liming Chen, Ping Jin, Fei Ma

**Affiliations:** 1 Laboratory for Comparative Genomics and Bioinformatics & Jiangsu Key Laboratory for Biodiversity and Biotechnology, College of Life Science, Nanjing Normal University, Nanjing 210046, Jiangsu, China; weiguanyun@gmail.com (G.W.); sunlianjey@gmail.com (L.S.); qinsjstu@gmail.com (S.Q.); leeramylrm@gmail.com (R.L.); jinping8312@163.com (P.J.); 2 School of Life Sciences, School of Ocean Nantong University, Nantong 226019, Jiangsu, China; 3 The Key Laboratory of Developmental Genes and Human Disease, College of Life Science, Nanjing Normal University, Nanjing 210046, Jiangsu, China; chenliming1981@njnu.edu.cn

**Keywords:** database, miRNA, disease, *Drosophila melanogaster*

## Abstract

Abnormal expressions of microRNA (miRNA) can result in human diseases such as cancer and neurodegenerative diseases. MiRNA mainly exert their biological functions via repressing the expression of their target genes. *Drosophila melanogaster* (*D. melanogaster*) is an ideal model for studying the molecular mechanisms behind biological phenotypes, including human diseases. In this study, we collected human and *D. melanogaster* miRNA as well as known human disease-related genes. In total, we identified 136 human disease-related miRNA that are orthologous to 83 *D. melanogaster* miRNA by mapping “seed sequence”, and 677 human disease-related genes that are orthologous to 734 *D. melanogaster* genes using the DRSC Integrative Ortholog Prediction Tool Furthermore, we revealed the target relationship between genes and miRNA using miRTarBase database and target prediction software, including miRanda and TargetScan. In addition, we visualized interaction networks and signalling pathways for these filtered miRNA and target genes. Finally, we compiled all the above data and information to generate a database designated DHDD This is the first comprehensive collection of human disease-related miRNA and their targeting genes conserved in a *D. melanogaster* database. The DHDD provides a resource for easily searching human disease-related miRNA and their disease-related target genes as well as their orthologs in *D. melanogaster*, and conveniently identifying the regulatory relationships among them in the form of a visual network.

## 1. Introduction

Over 3700 human genes with phenotype-causing mutations are identified and stored in the Online Mendelian Inheritance in Man database (OMIM database). Studies of human disease gene orthologs in model organisms have contributed to improved understanding of the molecular mechanisms of human disease [1]. Genome-wide analysis shows that approximately 70% of all known human disease-related genes have orthologous genes in *D. melanogaster* [2]. The major signal pathways involved in human disease are also evolutionarily conserved between human and *D. melanogaster*, for example, EGFR/RTK-Ras, PI3K, Notch, Wnt, Jak-STAT, Hedgehog and TGF-b pathways, which were first discovered from genetic studies of *D. melanogaster* [3,4,5,6]. In addition, body structures of adult *D. melanogaster* can perform equivalent functions to mammalian organs, such as the heart, kidney, lung, and reproductive system [7]. *D. melanogaster* is one of the most powerful models for studying the genetics and biology of human diseases [8,9,10,11], and is widely used as a model organism to study the pathogenesis of human diseases, such as cancer, neurodegenerative diseases, metabolic diseases [12,13,14,15], etc. The employment of *D. melanogaster* as a model system has successfully revealed the genetic and molecular mechanisms behind the process of neuronal degeneration, angiogenesis, innate immune response, stem cell selection and maintenance, cell and tissue polarity, signal transduction, growth control, behavioural neural control and organogenesis [11,14,16,17,18].

Identifying human gene orthologs in *D. melanogaster* is necessary to facilitate the use of *D. melanogaster* as the model system to study the function and mechanisms of human genes. Several databases, such as the Homophila database, the orthodisease database and the DIOPT-DIST database have been established [19,20,21], to provide valuable information for the further use of *D. melanogaster* as a model for the exploration of the molecular mechanisms of human diseases via cross-species genomic data analysis between human and *D. melanogaster*.

MiRNA play important roles in various human diseases, including cancers, cardiovascular diseases, neurodegenerative disorders, etc. [22,23,24,25,26]. In humans, miRNA are associated with diseases via regulation of the expression of disease-related genes. Many human disease-related miRNA databases are available, for examples, miR2Disease, The Human microRNA Disease Database [27,28,29,30]. Several databases, for example, miRGeneDB and miROrtho, provide information about orthologous miRNA between humans and *D. melanogaster*. However, no database provides information on the orthologs of human disease-related miRNA and their disease-related target genes in *D. melanogaster* to date.

To further facilitate the use of *D. melanogaster* as human disease model, in this study we constructed a Dme-Hsa disease database (DHDD) (http://bioinf.njnu.edu.cn/dhdd/home.php), an open web service, by identifying and analysing these orthologs of human disease-related miRNA and genes in *D. melanogaster*. In the DHDD, we provided information on human disease-related miRNA and genes and their orthologs in *D. melanogaster*. This database includes 83 miRNA and 734 genes of *D. melanogaster* that are homologous to 136 human miRNA and 677 human disease-related genes, respectively, as well as those related genes involved in 65 *D. melanogaster* pathways and 212 human pathways.

## 2. Database Description

### 2.1. Data Sources

Data were retrieved from miRBase (v22), Ensembl (release 89) and OMIM. In brief, mature sequences of the miRNA of *Homo sapiens* and *D. melanogaster* were downloaded from the miRBase (v22) database. Nucleotide sequences of *H. sapiens* were downloaded from Ensembl (release 89), and nucleotide sequences of *D. melanogaster* were downloaded from FlyBase (r6.08) [31,32,33], respectively. The list of disorders, disease genes, and associations among them was obtained from the Online Mendelian Inheritance in Man (OMIM) [34]. We classified these disease genes into 22 disorder classes based on their effects on different physiological systems [35].

### 2.2. Database Construction

**Figure 1:**
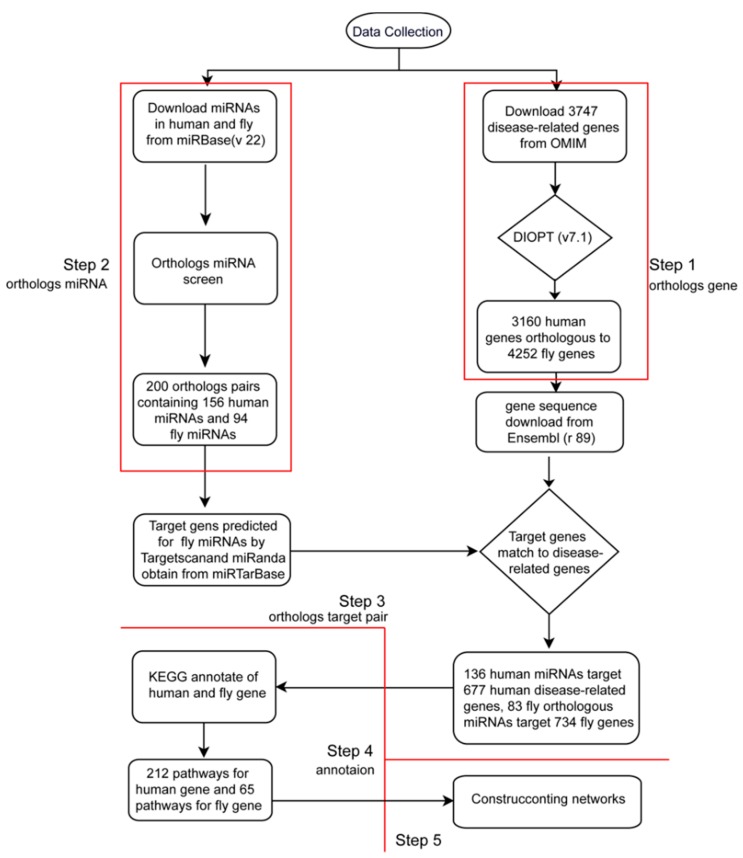
The analysis workflow of human disease-related miRNA and genes with orthologs in the fruit fly.

The workflow of the database construction is shown in Figure 1. In Step 1, we downloaded human disease-related genes from OMIM, and predicted their *Drosophila* orthologs using the DRSC Integrative Ortholog Prediction Tool (v7.1) (http://www.flyrnai.org.diopt). In Step 2, we downloaded human and *Drosophila* miRNA from miRBase (v22), then identified 156 human miRNAs with 94 orthologs of *D. melanogaster* miRNA by matching the seed sequences of miRNA (position 2–7 of 5′ end in mature miRNA) between human and *D. melanogaster*. In Step 3, we identified the orthologous pairs of miRNA to genes (OPMG). The process for identification of the OPMG was briefly as follows. Firstly, the 3′UTR of human and fly genes were download from Ensembl databse (release 89). Secondly, Targetscan (targetscan_70.pl) and miRanda (version 3.3a) were used to predict the regulation relationships between miRNA and genes in humans and *Drosophila*, respectively. The intersections of the predicted results of the two softwares were accepted, and the weak site type (6 mer) was filtered out. The experimentally validated relationships of miRNA regulating target genes were obtained from the miRTarBase, and then the intersections of the prediction and the experimental results were gained [36,37,38,39]. In Step 4, we used the KEGG database to annotate human and fly genes [40,41]. In Step 5, we used the Cytoscape program to visualize the OPMG pairs, pathways, and networks [42].

The DHDD database has been developed as a web page with ‘Home’, ‘Search’, ‘Browser’, ‘Download’ and ‘Help’ pages using an Apache HTTP web server and MySQL database server. The web page was constructed using the PHP language, and data was stored by MySQL. All data were processed using Perl language script. The networks were presented as pictures based on the analysis results. Queries from the ‘Search’ and ‘Browse’ pages retrieve the results from MySQL. All data in the DHDD database can be downloaded from the ‘Download’ page. Through the ‘Help’ page, users can get easily information on how to use our database and how to interpret the results of the search.

### 2.3. Database Content

#### 2.3.1. Identification of Orthologs of Human Disease-Related Genes in *D. melanogaster*

**Figure 2:**
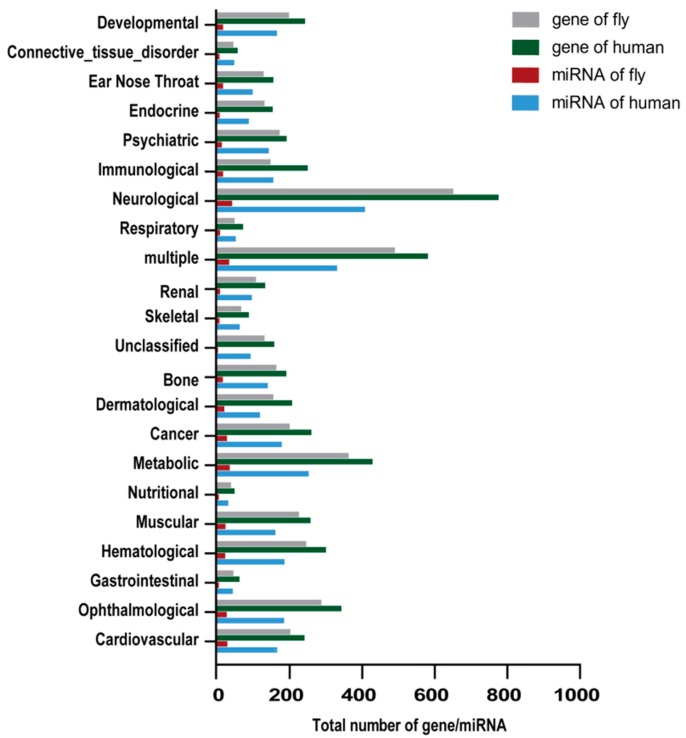
Statistical number of human disease-related genes and miRNA orthologs with the fly in different disease groups.

In total, 3747 human disease-related genes were retrieved from the OMIM morbid map and classified into 22 groups based on their involvement in physiological systems. The results showed that 3160 (84.33%) human disease-related genes had 4252 orthologs in *D. melanogaster*. The number of human disease-related genes and their *D. melanogaster* orthologs in different disease groups were calculated in Figure 2.

#### 2.3.2. Identification of Orthologs of Human Disease-Related miRNA in *D. melanogaster*

We identified 200 orthologous miRNA pairs containing 156 human miRNA and 94 *D. melanogaster* miRNA, which contribute to 13.52% and 8% of total miRNA number in human and *D. melanogaster*, respectively. The number of human miRNA target to disease-related genes and their orthologs in *D. melanogaster* were also calculated in Figure 2.

#### 2.3.3. Identification the Orthologous Pairs of miRNA to Gene (OPMG) in Human and *D. melanogaster*

We identified 4104 OPMG data entries, include 136 human miRNAs with orthologous to 83 *D. melanogaster* miRNAs, which respectively target 677 human disease-related genes and 734 orthologous genes in *D. melanogaster*. Remarkably, the gene orthologous pairs and miRNA orthologous pairs of *Drosophila* and human are not one-to-one, and the interaction in an OPMG may also be orthologous between human and fly.

#### 2.3.4. Pathway Annotation of Ortholog miRNA Target Genes between Human and *D. melanogaster*

In order to annotate the biological functions of OPMG, we analysed the genes with KEGG (Kyoto Encyclopedia of Genes and Genomes) annotations and identified 212 pathways for human disease-related genes and 65 pathways for *D. melanogaster* genes. The common pathways between human and *D. melanogaster* include Wnt, mTOR, Hippo, MAPK, TGF-beta, FoxO, and the Jak-STAT signaling pathway, etc. It is worth noting that the large number of genes from both human and *D. melanogaster* were annotated to be involved in ‘metabolic pathways’.

#### 2.3.5. Construction of Visual Networks

**Figure 3:**
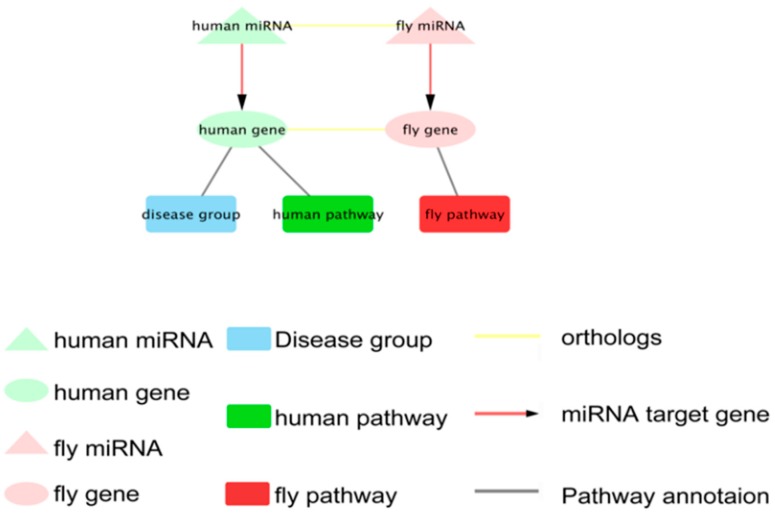
Example diagram of a network. Oval nodes represent genes, triangle nodes represent miRNA, squares nodes represent disease groups and pathways. Orthologous miRNA or genes are linked with a yellow line; miRNA are connected to genes by red lines with an arrow; genes are linked to pathways by gray lines.

The network of each disease group, human pathway, *Drosophila* pathway, human miRNA and their target genes as well as the orthologs in *D. melanogaster* were constructed based on the OPMG analysis. Based on the disease group and pathway, we constructed 22 networks of disease groups, including 216 networks of human pathways and 60 networks of *D. melanogaster* pathways in total. There are four types of nodes (gene, miRNA, disease group and pathway) and three types of edges (miRNA-target gene, orthologous and gene pathway annotation). Figure 3 shows an example diagram of a network. All of these networks were presented in the DHDD database as a dynamic network. At present, using *D. melanogaster* as a model organism to study human disease-related genes is mainly concentrated in the two groups ‘cancer’ and ‘neurological’ disease [43,44,45]. Among these networks, the ‘neurological’ network has 25 human genes and 27 *D. melanogaster* genes, and the ‘cancer’ network has 15 human genes and 16 *D. melanogaster* genes. In addition, the number of genes/miRNA in each network are listed in Table 1.

#### 2.3.6. Web Server Introduction and Use

**Figure 4:**
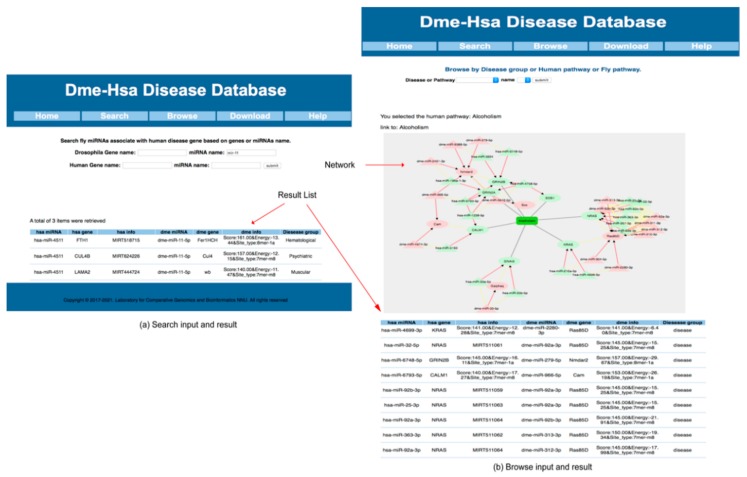
Web user interface, (**a**) on the search page, after entering a keyword, the result will be displayed and the list of the results will be displayed below; (**b**) On the browse page, select one group to display, the result is similar as on the search page. Both on the search and browse page, each row of the list is composed of five columns, human miRNA, fly miRNA, human gene, fly gene, gene disease group, where the human miRNA is orthologous to the fly miRNA, the human gene is orthologous to the fly gene, and the miRNA target to gene in human and fly, respectively.

On the search page of the DHDD database, the user can conveniently retrieve information (gene, miRNA and pathway) using keywords, such as the gene symbol and miRNA name (Figure 4a). For example, on the search web page, if the user searches the keyword “fly miRNA name: mir-11”, a return list includes *Drosophila* miRNA and genes, human miRNA and genes, the type of disease associated with mir-11, as well as the information on targeting relationships. Whilst the information on targeting relationships can be classified into two types, acquired from the miRTarbase (link to miRTarbase) or the results predicted by the software, and the database presents the miRTarbase ID (there could also be a link to the miRTarbase or the scores, energy and site type for target prediction). In addition, the human genes are also linked to the Ensembl database, the *Drosophila* genes are linked to the FlyBase database, and all miRNA are linked to the miRBase database. On the browse page, the user can easily retrieve information from the OPMG based on different categories (disease group, human pathway or fly pathway) (Figure 4b). The retrieval results include all OPMG information under this category and provides a visual network. The help page provides more details about the database. In addition, the download page can download all the data in the DHDD database.

## 3. Discussion and Conclusions

In the present study, our results show that many human disease-related genes have corresponding orthologous genes in *D. melanogaster*, and human disease-related genes can be regulated by orthologous miRNA between human and *D. melanogaster* (Figure 2). We identified the relationship of miRNA-regulating target genes that are conserved between human and *D. melanogaster*. In addition, we found that these miRNAs regulating target genes are classified into 22 disease groups, and 212 human pathways, as well as 65 fly pathways.

*Drosophila* is widely used as a feasible model organism in the study of human diseases [2,37,46]. Moreover, human miRNA was found to be involved in cancers, cardiovascular and neurological disease and so on [23,47,48]. Recent works have revealed that *D. melanogaster* is an ideal model organism to study human miRNA-related diseases, such as miR-124, which is involved in Alzheimer’s disease, and miR-219, which results in neurofibrillary degeneration [49,50,51]. Therefore, the importance of using *D. melanogaster* as a model to study the miRNA roles in human disease process is conspicuous. At present, the study of human disease using *D. melanogaster* as a model organism mainly is focused on the function of the disease-related genes. Although some studies have considered the regulatory role of miRNA during disease progression, large-scale analysis of the regulation relationship between miRNA and disease genes across different species is still limited [26,51]. The DHDD database provides very important resources for the investigation of the target relationship of human miRNA regulating human disease-related genes and their *D. melanogaster* orthologs. For example, *hsa*-miR-216a has been reported to be involved in pancreatic cancer progression, and a significant downregulation of hsa-miR-216a in human pancreatic cancer tissue has also been reported [52,53], which is consistent with our result in the DHDD database, indicating that the results from the DHDD database are reliable. In particular, in currently existing database resources, either only cross-species orthologous gene data or only human miRNA related to disease can be acquired. Therefore, our present work has remedied the limitations of these existing databases by providing valuable resources for the study of the role of miRNA in human diseases using *D. melanogaster* as a model.

In conclusion, the DHDD database can present human disease-related miRNA and orthologs in *D. melanogaster*, and a visual network can also conveniently show the regulation relationship between the miRNA and target genes, as well as providing more information resources in comparison to previous database resources.

## Figures and Tables

**Table 1:** The different nodes in all disease sub-networks.

Disease	Human		Fly	
	gene	miRNA	gene	miRNA
Bone	10	35	11	13
Cancer	15	54	16	31
Cardiovascular	6	10	5	7
Connective tissue disorder	5	20	7	13
Dermatological	3	10	3	10
Developmental	2	3	3	3
Ear Nose Throat	6	25	7	11
Endocrine	5	14	7	7
Gastrointestinal	2	4	4	4
Hematological	2	3	2	2
Immunological	9	31	9	12
Metabolic	8	33	7	11
Muscular	11	37	14	17
Neurological	25	55	27	36
Nutritional	1	1	1	1
Ophthalmological	9	25	11	21
Psychiatric	8	19	8	11
Renal	6	7	6	6
Skeletal	1	5	1	2
Unclassified	4	19	4	15
multiple	28	53	30	31
